# Simple mix-and-read assays for the determination of baclofen in pharmaceutical formulation

**DOI:** 10.1016/j.heliyon.2019.e01854

**Published:** 2019-06-07

**Authors:** Amira F. El-Yazbi, Karin M. Guirguis, Mona M. Bedair, Tarek S. Belal

**Affiliations:** a Pharmaceutical Analytical Chemistry Department, Faculty of Pharmacy, University of Alexandria, Elmessalah, 21521, Alexandria, Egypt; b Pharmaceutical Chemistry Department, Faculty of Pharmacy and Drug Manufacturing, Pharos University in Alexandria, Canal El-Mahmoudia Street, Alexandria, Egypt

**Keywords:** Organic chemistry, Analytical chemistry, Pharmaceutical science, Baclofen, Spectrophotometric determination, Vanillin, Eosin, Hantzsch reaction

## Abstract

This study demonstrates three simple and inexpensive spectrophotometric mix-and-read assays for the determination of the skeletal muscle relaxant, baclofen (BAC) in pharmaceutical formulations. The proposed methods are based on the reaction of the primary amine group of BAC with various derivatizing reagents to yield different colored products. Method I depends on the reaction of BAC with vanillin in borate buffer pH 11.5 to give a yellow colored product with maximum absorbance at 401 nm. While method II describes the reaction with eosin Y in citric-phosphate buffer pH 2.2 forming an orange-red product measured at 548 nm. Method III depends on Hantzsch condensation reaction that yields a yellow product measured at 339 nm. Different experimental variables influencing development and stability of the obtained colored product are optimized. Validation of the three methods regarding linearity, ranges, precision, accuracy and limits of detection and quantification was performed. Regression analysis showed good linearity over the concentration ranges of 10–35, 5–20 and 5–25 μg/mL for methods I, II and III, respectively with correlation coefficient values not less than 0.999. Additionally, detection limits of BAC are 1.58, 0.94 and 0.79 μg/mL for methods I, II and III, respectively. Finally, the suggested procedures are successfully used for assay of BAC in its tablets. The main advantages of the proposed mix-and-read assays are being inexpensive and rapid with no requirement for laborious extraction techniques with equivalent or superior sensitivity compared to other published spectrophotometric procedures. Such advantages promote the suggested methods for the high throughput assay of BAC dosage forms, a critical component in quality control studies for pharmaceutical industries.

## 1 Introduction

Baclofen (BAC) is a centrally acting muscle relaxant used in the management of spasticity. Chemically, BAC is *β*-(Aminomethyl)-4–chlorobenzenepropanoic acid. It may also act at supraspinal sites producing CNS depression [1].

Thorough literature survey revealed that chromatographic methods, mainly HPLC, are the mostly used methods for BAC analysis. Such as, HPLC with UV detection [2], determination in human plasma by HPLC with several detection methods including tandem mass spectrometry (MS-MS) [3, 4], high-resolution mass spectrometry (HR-MS) [5] and fluorescence detection [6, 7]. In addition, ultra-performance liquid chromatography coupled with MS-MS detection has been used for detecting BAC in blood samples [8]. On the other hand, capillary electrophoresis has been previously described for the determination of BAC in human serum using UV detection [9] or laser induced fluorescence detection [10]. Moreover, automated fluorimetric assay for BAC after on-line derivatization with o-phthalaldehyde [11] was described. Potentiometric determination was also used for the quantitative analysis of BAC in various dosage forms [12, 13]. Determination of BAC in biological fluids after derivatization with benoxaprofen chloride and TLC analysis was described [14]. Furthermore, a water-compatible superparamagnetic molecularly imprinted biopolymer was developed for clean separation of BAC from biological samples [15].

Direct spectrophotometry was also applied for determination of BAC as single component by measuring absorbance at 220 nm [16]. Some spectrophotometric methods exploited color-producing reactions based on the reaction with different chemical reagents such as ninhydrin [17, 18], sodium 1,2-napthoquinone-4-sulphonate [18], 4-chloro-7-nitro-2,1,3-benzoxadiazole [19], p-dimethyl amino benzaldehyde [20], ferric chloride followed by potassium ferric cyanide or 2,2-bipyridine [20] and 2,4-dinitrofluorobenzene [21], in addition to various derivative spectrophotometric methods [22]. Nevertheless, nearly all of the methods mentioned above need elaborate, laborious and complex sample pretreatment steps and lengthy cleaning procedure prior to the determination step. As a result, rapid, inexpensive and selective process is evidently needed for routine quality control assay of different pharmaceutical preparations.

The aim of this study is to design low-cost, rapid, simple and sensitive spectrophotometric techniques for the quantitation of BAC in pharmaceutical tablets with no requirement for laborious extraction processes. The study suggested is based on obtaining a colored product by the derivatization of BAC with vanillin in alkaline borate buffer (method I), eosin Y in acidic citric-phosphate buffer (method II) and acetylacetone/formaldhyde in acidic pH by Hantzsch condensation reaction (method III). A throughout literature survey indicated that the three selected techniques have not been previously applied for the determination of BAC in different tablet formulation. The proposed work offers ideal analytical methods for its routine analysis and quality assurance of pharmaceutical formulations, particularly in pharmaceutical laboratories where spectrophotometers are readily available for analysis.

## 2 Experimental

### 2.1 Instrumentation

Helios alpha Unicam UV-VIS spectrophotometer coupled to Version 32 computer system was used in this study for all the spectrophotometric measurements in a 1-cm quartz cells.

### 2.2 Materials and reagents

Baclofen (BAC) standard of 99.8 % purity was kindly supplied by Pharaonia Pharmaceuticals, Alexandria, Egypt. Vanillin (Sigma-Aldrich, St. Louis, MO, USA) and eosin Y (Sigma-Aldrich, St. Louis, MO, USA) were used. Analytical reagent grade including citric acid (El-Nasr Pharmaceutical Co., Cairo, Egypt), disodium phosphate (El-Nasr Pharmaceutical Co., Cairo, Egypt), borax (El-Nasr Pharmaceutical Co., Cairo, Egypt), sodium hydroxide (El-Nasr Pharmaceutical Co., Cairo, Egypt), formaldehyde (El-Nasr Pharmaceutical Co., Cairo, Egypt), acetylacetone (Research-Lab Fine Chem Industries, Mumbai, India), sodium acetate (El-Nasr Pharmaceutical Co., Cairo, Egypt), glacial acetic (El-Nasr Pharmaceutical Co., Cairo, Egypt), hydrochloric acid (El-Nasr Pharmaceutical Co., Cairo, Egypt) and methanol (Sd fine-chem limited, Mumbai, India) were utilized. Baclofen^®^ tablets (Misr Company for Pharmaceuticals, Cairo, Egypt, batch no. 107016) and Mylobac^®^ tablets (Pharaonia Pharmaceuticals, Alexandria, Egypt, batch no. 1205007), both labeled to contain 10 mg of BAC per tablet, were used.

### 2.3 Preparation of reagents

#### 2.3.1 Method I

3% vanillin solution was prepared by dissolving 0.75 g vanillin in 25 mL of methanol. While borate buffer (pH 11.5) was prepared by mixing a volume of 50.0 mL of 0.05 M borax with 45.0 mL of 0.1 M sodium hydroxide then complete to 100.0 mL with distilled water.

#### 2.3.2 Method II

Eosin Y aqueous solution (0.26 %) consisted of 0.26 g eosin Y in 100.0 mL distilled water. While citric – phosphate buffer (pH 2.2) was composed of 98.0 mL of 0.1 M citric acid with 2.0 mL of 0.2 M disodium phosphate.

#### 2.3.3 Method III

The derivatizing reagent was prepared in a test tube by mixing 3.0 mL of distilled water, 0.5 mL acetate buffer (pH 4), 0.5 mL formaldehyde and 1.0 mL acetylacetone. Excess drops of formaldehyde were added to obtain clear solution. The reagent should be freshly prepared.

### 2.4 Preparation of standard solutions

BAC standard stock solutions of 500.0 μg/mL (method I) and 250.0 μg/mL (method II and III) were prepared in distilled water (method I and II) and in methanol (method III). All solutions were kept in a refrigerator at 4 °C.

### 2.5 General procedures

#### 2.5.1 Method I

Exact volumes (0.20–0.70 mL) of BAC solution (500.0 μg/mL) were transferred into a set of 10 mL-volumetric flasks. A volume of 0.80 mL of 3% vanillin was added followed by 0.40 mL of borate buffer (pH 11.5). Solutions were heated at 50 °C for 20 min, cooled, then completed to volume with methanol. Finally, the absorbance of the yellow colored product was measured at 401 nm against reagent blank.

#### 2.5.2 Method II

Exact volumes (0.20–0.80 mL) of BAC solution (250.0 μg/mL) were transferred into a set of 10 mL-volumetric flasks, completed to 6.00 mL with distilled water. 2.25 mL of 0.26% eosin Y aqueous solution was added to each flask. The solutions were mixed well and 0.40 mL citric-phosphate buffer pH 2.2 was added, then completed to volume with distilled water. Finally, the absorbance of the orange-red colored product was obtained at 548 nm against reagent blank.

#### 2.5.3 Method III

Working solutions of BAC were prepared by mixing different volumes of the stock solution (0.20–1.00 mL) with 0.4 mL of derivatizing reagent into screw-capped tubes, followed by heating at 90 °C for 10 min in a thermo-stated water bath. After cooling, solutions were transferred into 10 mL volumetric flasks and completed to volume with distilled water. The formed yellow products were measured at 339 nm against reagent blank.

For each method, the calibration graph was constructed by plotting the obtained absorbance readings against the corresponding concentrations.

### 2.6 Analysis of pharmaceutical tablets

Eight tablets from each formulation were separately weighed and finely powdered. Accurately weighed portions of the powder equivalent to 25.0 mg BAC for method I and 12.5 mg BAC for method II and III were separately extracted with 20 mL distilled water (for method I and II) or methanol (for method III) by votexing for 10 min then filtered into 50 mL volumetric flasks. The residue was washed with 2 × 10 mL portions of distilled water in method I, II and methanol in method III. Washings were added to the filtrate, and finally completed to volume with distilled water (method I, II) and methanol (method III) to reach a final concentration 500 μg/mL of BAC for method I and 250 μg/mL of BAC for method II and III. Finally, analysis was carried out using the same procedures as the general procedures described above. Recovery values were calculated using standard solutions measured using the same procedure.

## 3 Results and discussion

### 3.1 Absorption spectral studies

**Fig. 1:**
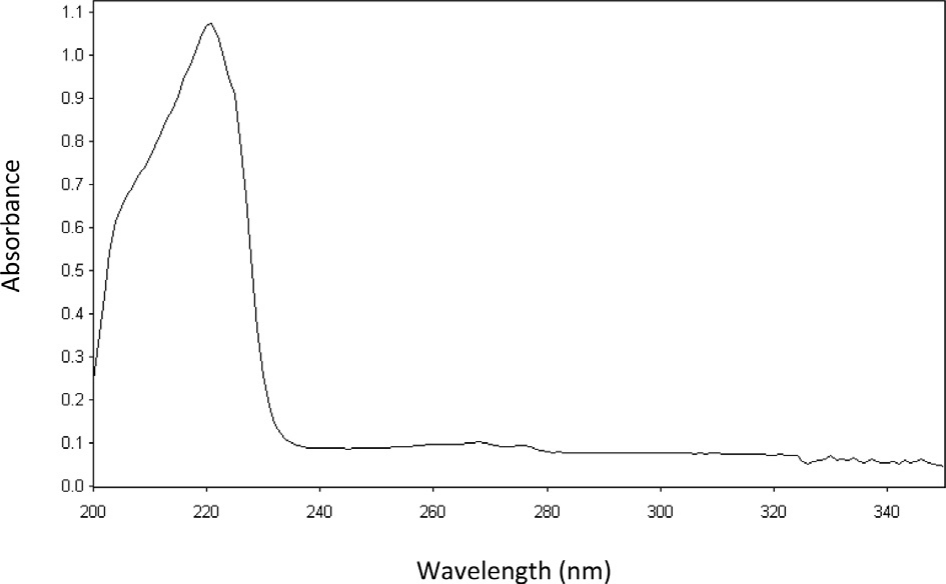
Absorption spectrum of 20 μg/mL BAC in methanol.

**Scheme 1:**
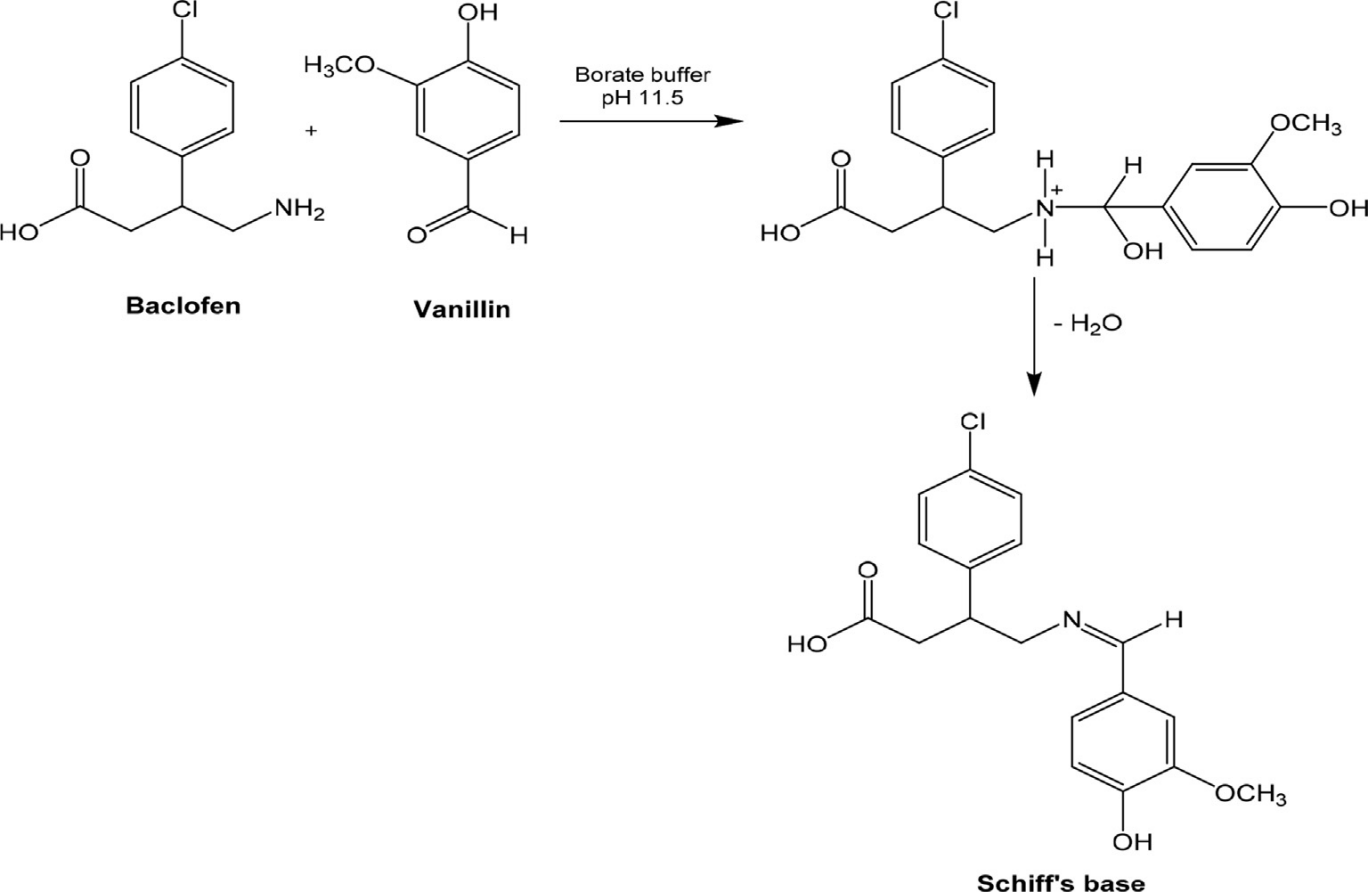
Pathway proposed for the reaction of BAC with vanillin.

**Fig. 2:**
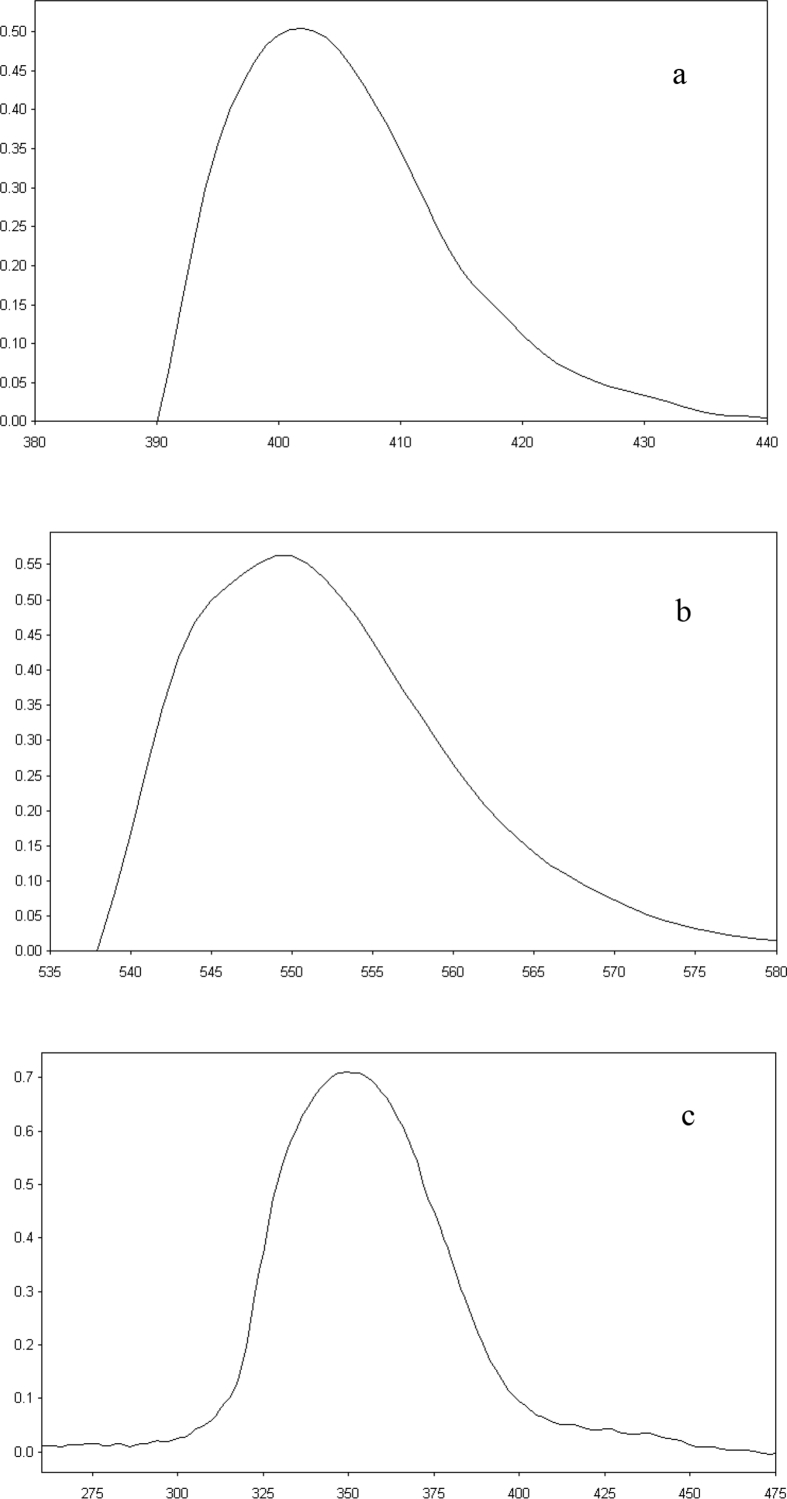
Absorption spectra of the reaction products of (a) 30 μg/mL BAC with vanillin (method I) with λ_max_ at 401 nm, (b) 20 μg/mL BAC with eosin Y (method II) with λ_max_ at 548 nm and (c) 15 μg/mL BAC using Hantzsch Reaction (method III) with λ_max_ at 339 nm.

BAC shows absorption in the short UV region showing an absorption maximum at 220 nm (Fig. 1). In this study, we developed three spectrophotometric assays for the estimation of BAC in bulk form and in tablets dosage forms. The suggested methods are based on the reaction of the primary amine group in BAC with three different reagents; vanillin, eosin Y and Hantzsch reaction, resulting in the formation of intense colored products. Method I is based on the condensation of BAC with vanillin to form Schiff's base. The Schiff's base is formed via the reaction of the aromatic amine in BAC with the aldehydic group in vanillin through condensation reaction [23] (Scheme 1). The yellow colored product absorbed maximally at 401 nm (Fig. 2a).

**Scheme 2:**
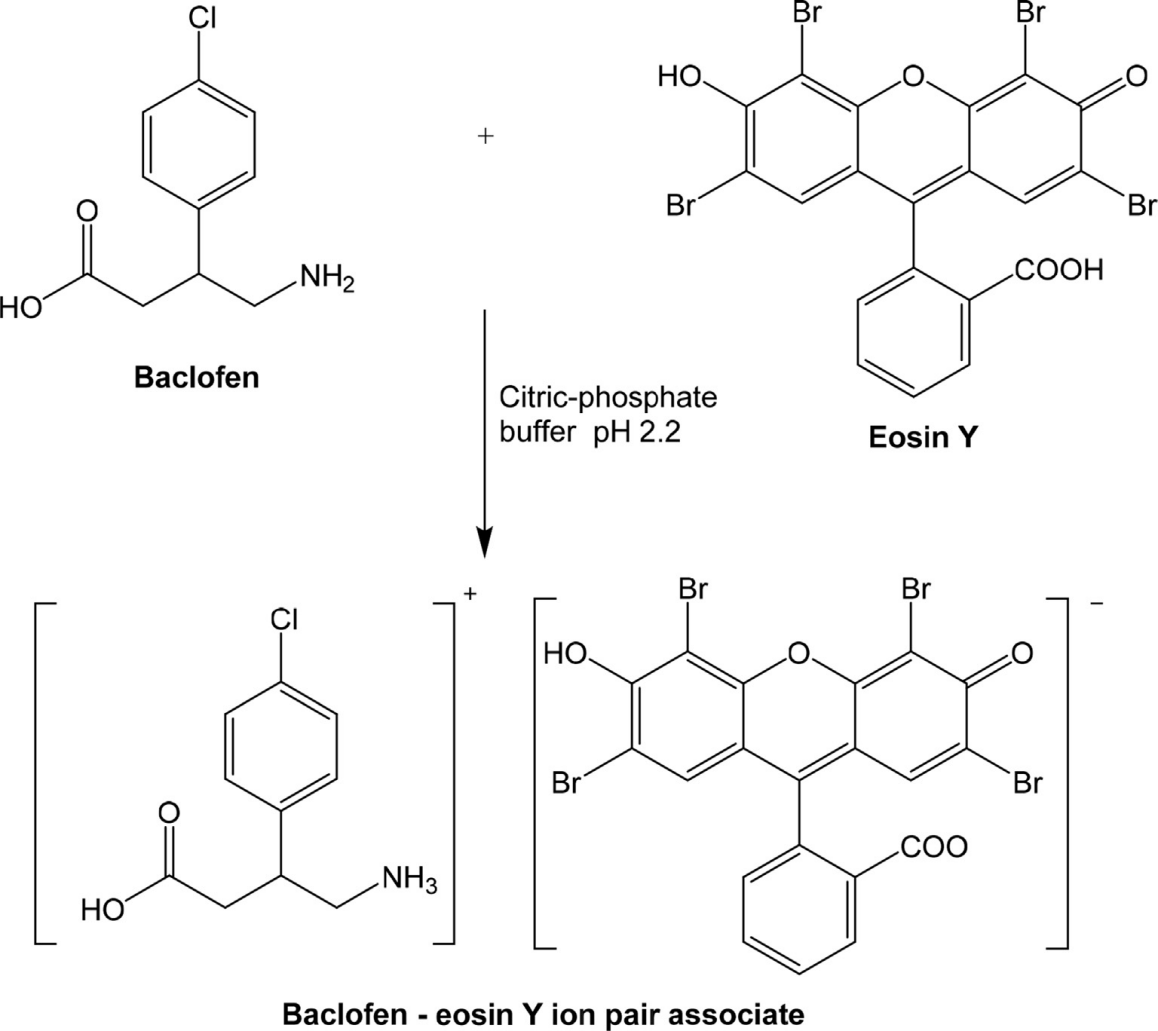
Pathway proposed for the reaction of BAC with eosin Y.

While method II depends on the complexation of BAC with a xanthene dye, eosin Y, through electrostatic attraction between the protonated terminal amino group of BAC and the carboxylic group of eosin Y [24, 25] forming an ion-pair with BAC in acidic medium with an absorption maximum at 548 nm (Fig. 2b). Scheme 2 demonstrates the proposed mechanism for this reaction.

**Scheme 3:**
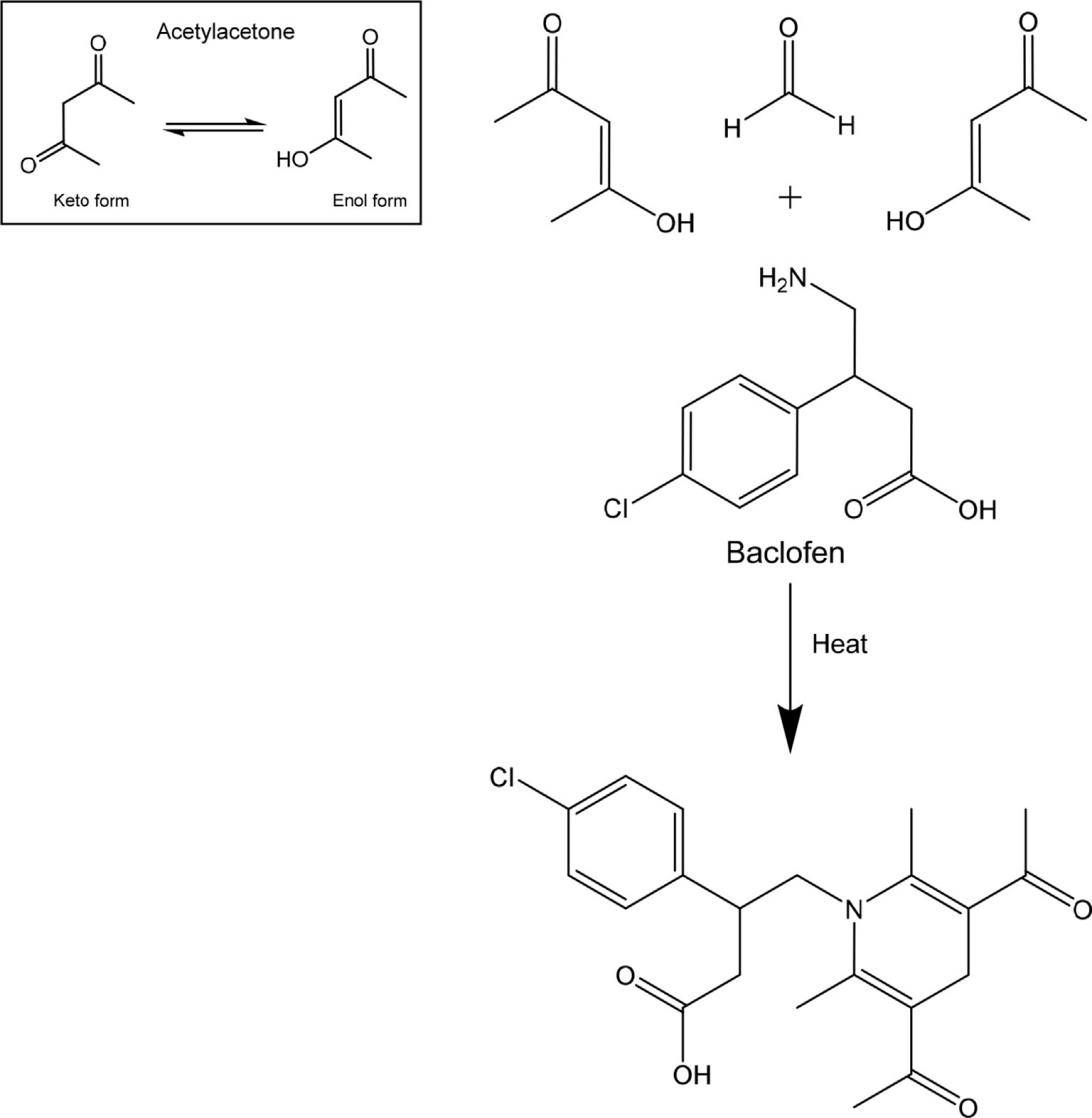
Pathway proposed for Hantzsch condensation reaction of BAC with acetylacetone and formaldehyde.

For method III, it is based on Hantzsch condensation reaction [26, 27] where BAC reacts with a β-dicarbonyl compound (acetylacetone) and an aldehyde (formaldehyde) to generate a yellow colored product with λ_max_ at 339 nm (Fig. 2c). The reaction mechanism is suggested in Scheme 3.

### 3.2 Optimization of the experimental conditions

Several experimental conditions were studied and optimized in order to obtain maximum and reproducible absorbance results. These factors include: reagent concentration, buffer (type, pH and volume) used, diluting solvent, reaction temperature and reaction time.

#### 3.2.1 Method I

##### 3.2.1.1 Reagent concentration

**Fig. 3:**
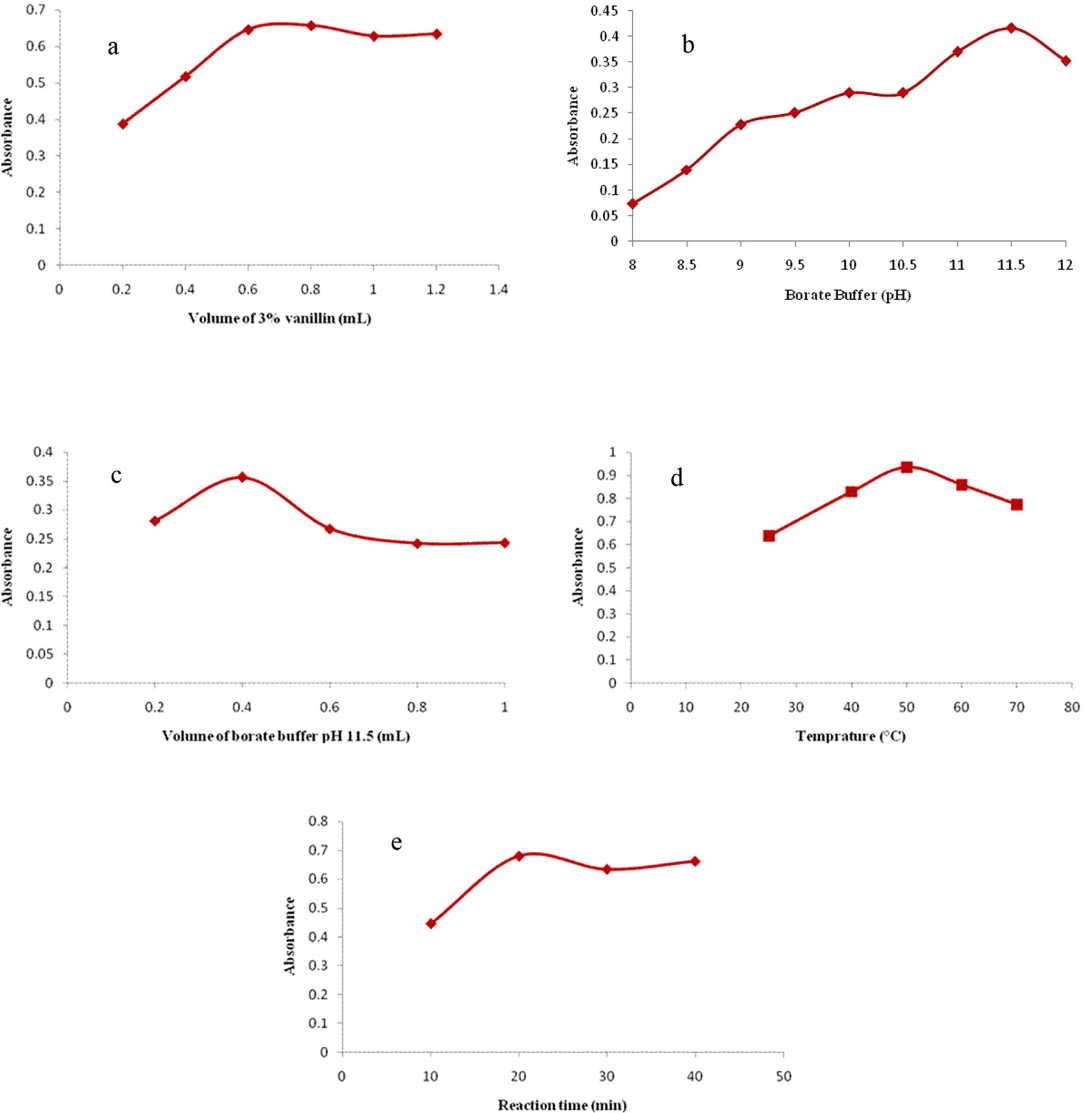
Optimization of experimental conditions for the reaction of 50 μg/mL BAC with 3% vanillin (Method I) recorded at 401 nm (a) effect of volume of vanillin (with optimum value of 0.80 mL), (b) effect of borate buffer pH (with optimum value of pH 11.5), (c) effect of borate buffer volume (with optimum value of 0.40 mL), (d) effect of the reaction temperature (with optimum value of 50 °C) and (e) effect of heating time (with optimum value of 20 min) on the absorbance of the colored product.

Different volumes of 3% vanillin were examined in order to study the effect of reagent concentration while keeping the other parameters constant at: 0.40 mL borate buffer of pH 11.5, reaction temperature at 50 °C and heating time for 30 min. It was found that 0.80 mL of vanillin was adequate to reach the maximum color intensity, after which no more raise in absorbance was achieved (Fig. 3a).

##### 3.2.1.2 Buffer type, pH and volume

Several trials using aqueous hydrochloric acid, glacial acetic acid and phosphate buffer pH 6 were performed and were unsuitable showing no absorption peaks. However, upon examining borate buffer with basic pH, yellow color was obtained. As the borate buffer pH increased, the color intensity increased. Maximum absorbance was observed at pH 11.5 above which a decrease in absorbance was recorded (Fig. 3b). Regarding buffer volume, it was found that 0.40 mL of borate buffer pH 11.5 was sufficient to reach maximum color intensity (Fig. 3c). Tested solutions were heated at 50 °C for 30 min using 0.80 mL of vanillin.

##### 3.2.1.3 Reaction temperature

To select the most appropriate reaction temperature while heating for 30 min using 0.40 mL of borate buffer pH 11.5 and 0.80 mL of vanillin, the reaction was carried out at different temperatures (room temperature, 40, 50, 60 and 70 °C). 50 °C was considered as the optimal temperature for the reaction of BAC with vanillin (Fig. 3d).

##### 3.2.1.4 Heating time

Heating time was optimized to reach the best sensitivity by recording the color developed at 50 °C for various time intervals. Maximum color intensity was achieved after 20 min (Fig. 3e) with heating at 50 °C temperature using 0.40 mL of borate buffer pH 11.5 and 0.80 mL of vanillin.

#### 3.2.2 Method II

##### 3.2.2.1 Buffer type, pH and volume

**Fig. 4:**
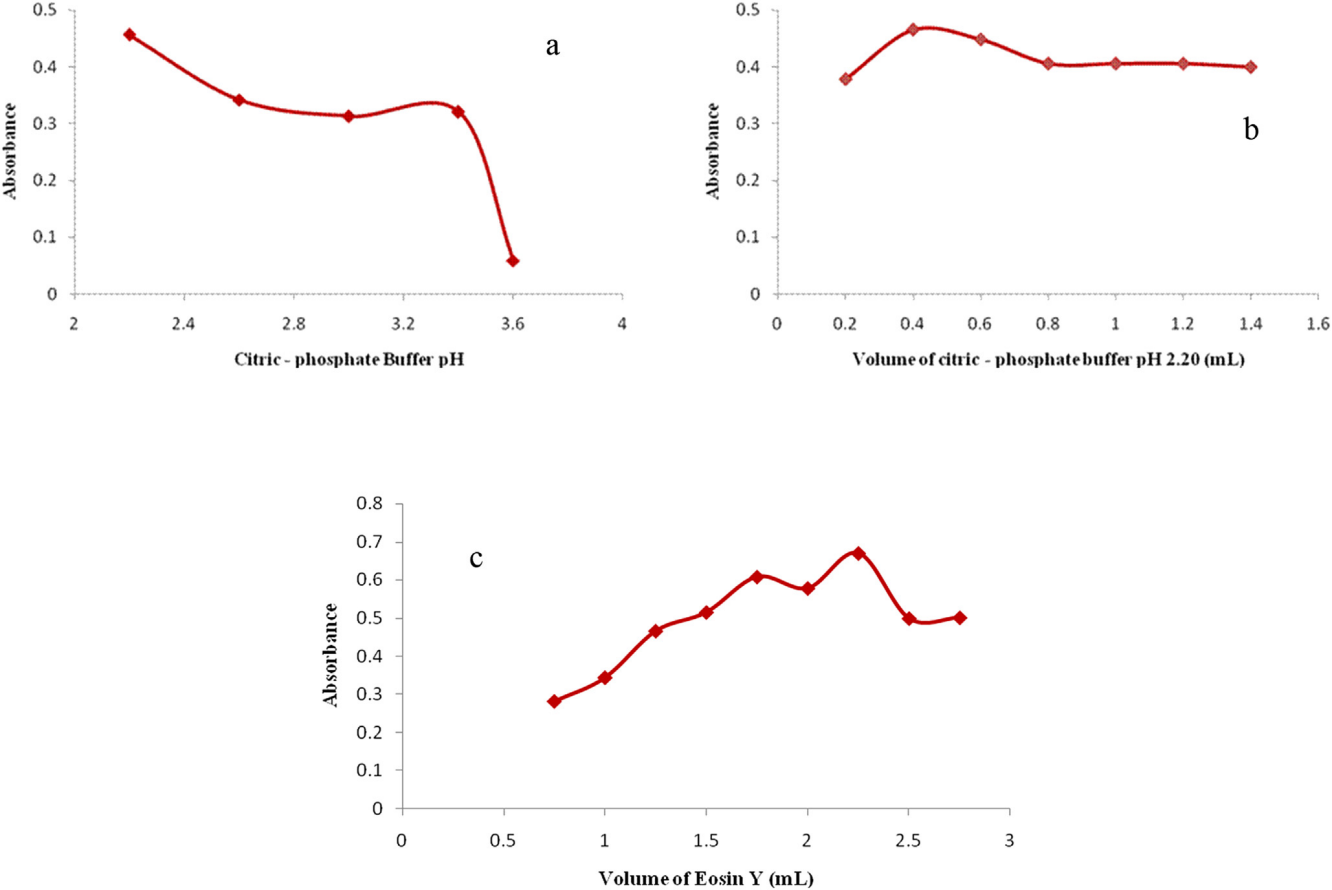
Optimization of experimental conditions for the reaction of 20 μg/mL BAC with 0.26% eosin Y (Method II) recorded at 548 nm (a) effect of pH of citric-phosphate buffer (with optimum value of pH 2.20), (b) effect of volume of citric-phosphate buffer pH 2.20 (with optimum value of 0.40 mL) and (c) effect of the volume of eosin Y (with optimum value of 2.25 mL) on the absorbance of the reaction product.

Several trials using Walpole's acetate buffer (0.20 M) pH 3.6 were performed. However, lower pH could not be reached with Walpole's acetate buffer, therefore, citric-phosphate buffer (Mclivaine's Buffer) was used. Different pH values in the range 2.20–3.80 were tried while using 2.25 mL of 0.26 % eosin Y solution. Results show that 0.40 mL of citric-phosphate buffer pH 2.20 was adequate to attain the maximum color intensity, after which no more rise in absorbance was detected. Fig. 4a and b demonstrate the effect of buffer pH and volume on the measured absorbance.

##### 3.2.2.2 Reagent concentration

The effect of reagent concentration was investigated by examining different volumes of stock eosin Y solution (0.26 %) added. A volume of 2.25 mL of eosin Y in 0.40 mL of citric-phosphate buffer pH 2.20 was found to be adequate to reach the highest color intensity. The effect of concentration on absorbance of the produced color product is demonstrated in Fig. 4c.

#### 3.2.3 Method III

##### 3.2.3.1 Reagent concentration

**Fig. 5:**
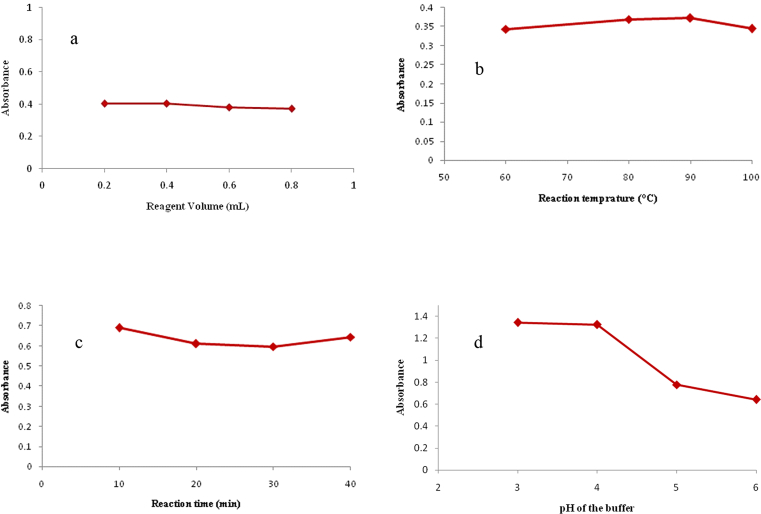
Optimization of experimental conditions for the reaction of 20 μg/mL BAC in Hantzsch condensation reaction (Method III) recorded at 339 nm (a) effect of volume of derivatizing reagent (with optimum value of 0.40 mL), (b) effect of reaction temperature with optimum value of 90 °C), (c) effect of reaction time (with optimum value of 10 min) and (d) effect of the pH of acetate buffer (with optimum value of pH 4) on the absorbance of the colored product.

Different volumes of the derivatizing reagent were examined in order to study the influence of reagent concentration while performing the reaction at 90 °C for 10 min and completing to volume with distilled water. Results show that 0.40 mL reagent was adequate to obtain the optimum color intensity; thereafter no more rise in absorbance was indicated (Fig. 5a).

##### 3.2.3.2 Reaction temperature

The reaction temperature was optimized to reach the best sensitivity by monitoring the color development at different temperatures (60°, 80°, 90° and 100 °C) while heating for 10 min and completing to volume with distilled water. Increasing the temperature showed slight increase in the measured absorbance to reach its maximum at 90 °C, therefore it was chosen for as optimum temperature (Fig. 5b).

##### 3.2.3.3 Reaction time

Time factor was optimized by studying the development of the yellow color at 90 °C at several time intervals and completing to volume with distilled water. Complete color formation was achieved after 10 min with no further increase in absorbance (Fig. 5c).

##### 3.2.3.4 Buffer pH

The effect of different pH values of 0.1 M acetate buffer in the range of pH 3–6 were included in the preparation of the reagent. 0.40 mL of the reagent was added to the BAC, heated at 90 °C for 10 min, then completed to volume with distilled water. Results showed that pH range of 3–4 resulted in maximum absorption, therefore pH 4 was selected for the reaction (Fig. 5d).

##### 3.2.3.5 Diluting solvent

Different solvents were examined in order to select the most suitable diluting solvent, such as acetate buffer, methanol, acetonitrile, ethanol and distilled water. 0.40 mL of the reagent prepared in acetate buffer, pH 4 was added to the BAC, heated at 90 °C for 10 min, then completed to volume with the appropriate solvent. Results show that all solvents obtain almost the same intensity. Distilled water as the most ecofriendly solvent, was selected for the reaction.

### 3.3 Validation of the proposed methods

The suggested methods were validated following the ICH guidelines [28].

#### 3.3.1 Linearity and concentration ranges

Series of different concentrations of BAC were analyzed to assess the linearity of the proposed methods. Table 1 shows the performance data and linearity parameters for the proposed methods. Regression analysis for the calibration curves of the drug revealed good linear relationship throughout the concentration ranges of 10–35 μg/mL, 5–20 μg/mL and 5–25 μg/mL for methods I, II and III, respectively. The correlation coefficients for the assay of BAC using the proposed methods exceeded 0.999 with RSD% of slope values not exceeding 2%.

**Table 1:** Analytical parameters for the determination of BAC using the proposed spectrophotometric methods.

Parameter	Method I	Method II	Method III
Wavelength (nm)	401	548	339
Concentration range (μg/mL)	10–35	5–20	5–25
Apparent molar absorptivity (L mol^−1^ cm^−1^)	2756	5491	9935
Intercept (a)	0.1174	0.0393	0.0066
S_a_	0.0062	0.0073	0.0112
Slope (b)	0.0129	0.0257	0.0465
S_b_	0.000257	0.0005	0.0007
RSD% of the slope (S_b_%)	1.99	1.95	1.51
Correlation coefficient (r)	0.99921	0.99889	0.99968
S_y/x_	0.0054	0.0072	0.0107
F value	2520	2245	4723
Significant F	9.42 × 10^−7^	7.90 × 10^−8^	6.79 × 10^−6^
LOD (μg/mL)	1.58	0.94	0.79
LOQ (μg/mL)	4.79	2.84	2.41

#### 3.3.2 Limit of detection (LOD) and limit of quantitation (LOQ)

LOD and LOQ were defined as 3.3σ/S and 10σ/S respectively, where σ is the standard deviation of the intercept of regression line and S is the calibration graph slope. LOD values were 1.58, 0.94 and 0.79 μg/mL for methods I, II and III, respectively. While for the LOQ values, 4.79, 2.84 and 2.41 μg/mL were obtained for methods I, II and III, respectively. Evidently, the LOD, LOQ and molar absorptivity (ɛ) values (Table 1) highlight that the Hantzsch reaction (method III) gives the best sensitivity for the determination of BAC among the three suggested methods.

#### 3.3.3 Accuracy and precision

Three replicate determinations for each concentration of three different concentration levels of BAC (15, 25 and 35 μg/mL for method I and 10, 15 and 20 μg/mL for methods II and III) were examined in order to assess the accuracy and within-day precision for the suggested methods. Moreover, the between-day precision were performed as within-day precision but on three different days. The recovered concentrations were assessed using the corresponding regression equations. The analytical results acquired are demonstrated in Table 2. The percentage relative standard deviation (RSD %) and the percentage relative error (Er %) values were less than 2% demonstrating the high precision and good accuracy of the suggested methods.

**Table 2:** Precision and accuracy for the determination of BAC in bulk form using the proposed spectrophotometric methods.

Method		Nominal value (μg/mL)	Found ± SD^a^ (μg/mL)	RSD (%)^b^	E_r_ (%)^c^
Method I	Within-day	15	14.71 ± 0.23	1.56	–1.93
		25	25.34 ± 0.15	0.59	01.36
		35	35.21 ± 0.32	0.91	00.60
	Between-day	15	14.96 ± 0.30	2.01	–0.27
		25	25.08 ± 0.07	0.28	00.32
		35	34.97 ± 0.60	1.72	–0.09
Method II	Within-day	10	10.05 ± 0.09	0.90	00.50
		15	15.16 ± 0.17	1.12	01.07
		20	20.16 ± 0.33	1.64	00.80
	Between-day	10	10.07 ± 0.15	1.49	00.70
		15	14.96 ± 0.28	1.87	–0.27
		20	20.34 ± 0.38	1.87	01.70
Method III	Within-day	10	09.92 ± 0.03	0.30	–0.80
		15	15.20 ± 0.15	0.99	01.33
		20	19.59 ± 0.16	0.82	–2.05
	Between-day	10	9.97 ± 0.02	0.20	–0.30
		15	14.91 ± 0.17	1.14	–0.60
		20	19.82 ± 0.04	0.20	–0.90

a Mean ± standard deviation for three determinations.

b % Relative standard deviation.

c % Relative error.

### 3.4 Assay of tablets dosage forms

Analysis of BAC in its tablet dosage forms; Mylobac® and Baclofen® tablets were established using the spectrophotometric assays proposed in this study. The reported excipients for Baclofen® tablets are: lactose monohydrate, magnesium stearate, povidone K 25, crospovidone, sodium starch glycolate type A. While for Mylobac® tablets, the reported excipients are lactose monohydrate, maize starch, pregelatinised starch, croscarmellose sodium, colloidal silicon dioxide, magnesium stearate and microcrystalline cellulose. Recoveries were calculated using external standard method. The analysis results showed satisfactory accuracy and precision as revealed from % found, SD and RSD% values (Table 3) without any interference from the excipients present in both dosage forms. The good recoveries revealed the lack of any interference from commonly added excipients.

**Table 3:** Application of the proposed spectrophotometric methods for the determination of BAC in commercial tablets.

Mylobac® tablets	(Method I)	(Method II)	(Method III)	Reported method [29]
%Found ±SD^∗^	97.50 ± 0.26	97.91 ± 1.20	97.47 ± 0.72	97.05 ± 0.48
RSD%	0.27	1.23	0.74	0.49

ANOVA (single factor)						
Source of Variation	SS	df	MS	F	P-value	F critical
Between Groups	1.825975	3	0.608658	**1.08756**	0.382722	**3.238872**
Within Groups	8.95448	16	0.559655			
Total	10.78046	19				

Baclofen® tablets	(Method I)	(Method II)	(Method III)	Reported method [29]
%Found ±SD^∗^	99.37 ± 0.57	98.94 ± 0.41	98.55 ± 1.30	98.64 ± 0.92
RSD%	0.57	0.41	1.32	0.93

ANOVA (single factor)						
Source of Variation	SS	df	MS	F	P-value	F critical
Between Groups	2.026095	3	0.675365	**0.893133**	0.466005	**3.238872**
Within Groups	12.0988	16	0.756175			
Total	14.1249	19				

^∗^Mean ± standard deviation for five determinations.

Moreover, a simple reported HPLC method [29] was applied for assay of BAC in its tablets. The reported method was designed using C18 column (250 × 4.6 mm, 5μm) as stationary phase, acetonitrile/orthophosphoric acid pH 3.5 as mobile phase and UV detection at 220 nm. Recoveries acquired from the suggested methods were statistically compared with those of the reported method using one-way analysis of variance (Single factor ANOVA). The calculated F-value was not more than the critical value, thus, showing no significant differences between the proposed and the reported methods (Table 3). Such results suggest that the spectrophotometric methods proposed in this study are suitable for BAC routine analysis in tablets available in the market with a satisfactory level of accuracy and precision.

### 3.5 Comparison with other methods

Literature reveals only few spectrophotometric methods for the estimation of BAC. Table 4 presents a comparison of the three suggested methods with other reported spectrophotometric methods for BAC analysis. Critical comparison shows that the suggested methods provide comparable sensitivity to other derivatization methods with the advantages of being simple inexpensive mix-and-read assays without any tedious multi-step procedures that may lead to sample loss. Also, being independent on expensive instrumentation or specific analytical reagents, in addition to applying water as the diluting solvent allows this method to be a greener alternative to some of the other published methods.

**Table 4:** Comparison with other reported spectrophotometric methods for BAC determination.

Parameters	Proposed spectrophotometric methods	Reported spectrophotometric methods
Analytical reagent	Method I: vanillin (Schiff's base reaction) Method II: Eosin (Complex formation reaction) Method III: Acetylacetone/formaldehyde (Hantzsch condensation reaction)	Direct spectrophotometric measurements (16) derivative spectrophotometric methods (zero order, first order) (22) ninhydrin reagent (17) NBD-Cl (19) A-Ninhydrin reagent, B- NQS reagent (18) FDNB (21) **Method 1:** p-dimethyl amino benzaldehyde, **method 2**: FeCl_3_/2,2,-bipyridine, **method 3**: FeCl_3_/potassium ferric cyanide (20)
λnm	Method I: 401nm Method II: 548 nm Method III: 339 nm	220 nm (16) zero order: 220 nm, first order: 215 nm (22) 570 nm (17) 465 nm (19) A- 403 nm, B- 555.5 nm (18) 355 nm (21) Method 1: 430 nm, method 2: 550 nm, method 3: 730 nm (20)
Linearity range	Method I: 10–35 μg/mL Method II: 5–20 μg/mL Method III: 5–25 μg/mL	10–100 μg/mL (16) 3–18 μg/mL (for both zero order and first order) (22) 0.4–1.4 mg/mL (17) 5–70 μg/mL (19) A- 20–60 μg/ml, B- 10–60 μg/ml (18) 1–15 μg/mL (21) Method 1: 5–70 μg/mL, method 2 and 3: 1–25 μg/mL (20)
LOD	Method I: 1.58 μg/mL Method II:0.94 μg/mL Method III: 0.79 μg/mL	1.25316 μg/mL (16) zero order: 0.039 μg/mL, first order: 0.952 μg/mL (22) 0.0035 mg/mL (17) LOD (×10^3^) = 0.152 (19)
LOQ	Method I: 4.79 μg/mL Method II: 2.84 μg/mL Method III: 2.41 μg/mL	3.797468 μg/mL (16) zero order: 0.120 μg/mL, first order: 2.886 μg/mL (22) LOQ (×10^3^) = 0.510 (19)

## 4 Conclusion

Three simple, eco-friendly, inexpensive and rapid spectrophotometric methods were proposed for the determination of the muscle relaxant drug, baclofen in bulk form and in tablets dosage forms. The proposed methods took advantage of the presence of the primary amine group in the drug to react with several reagents including vanillin, eosin Y and acetyl acetone/formaldehyde reagents for the formation of intense color products. Good assay results were obtained upon application of the described methods on baclofen dosage forms, and the results were comparable to a reported HPLC assay. The proposed methods are simple inexpensive mix-and-read assays for the determination of baclofen that are suitable for the high throughput analysis of baclofen in its dosage forms.

## Declarations

### Author contribution statement

Amira El-Yazbi, Tarek Belal: Conceived and designed the experiments; Analyzed and interpreted the data; Contributed reagents, materials, analysis tools or data; Wrote the paper.

Karin Guirguis: Performed the experiments; Analyzed and interpreted the data; Contributed reagents, materials, analysis tools or data; Wrote the paper.

Mona Bedair: Conceived and designed the experiments; Contributed reagents, materials, analysis tools or data.

### Funding statement

This research did not receive any specific grant from funding agencies in the public, commercial, or not-for-profit sectors.

### Competing interest statement

The authors declare no conflict of interest.

### Additional information

No additional information is available for this paper.
